# Inhibiting the Musculoskeletal Pathological Processes in Post-knee Replacement Surgery With Osteopathic Manipulative Treatment: A Systematic Review

**DOI:** 10.7759/cureus.21599

**Published:** 2022-01-25

**Authors:** YaQun Zhou, Justin Chin, Abigail Evangelista, Blake Podger, Peter J Wan, Christine M Lomiguen

**Affiliations:** 1 Department of Surgery, CarePoint Health Hoboken University Medical Center, Hoboken, USA; 2 Department of Medical Education, Lake Erie College of Osteopathic Medicine, Erie, USA; 3 Department of Family Medicine, LifeLong Medical Care, Richmond, USA; 4 Department of Anatomy, Touro College of Osteopathic Medicine, New York City, USA; 5 Department of Internal Medicine, Northwell Health Lenox Hill Hospital, New York City, USA; 6 Department of Family Medicine, Millcreek Community Hospital, Erie, USA

**Keywords:** knee osteoarthritis (koa), tka, total knee arthroplasty, physical therapy, oa, knee osteoarthritis, osteoarthritis, osteopathic medicine, osteopathic manipulative medicine (omm), total knee replacement (tkr)

## Abstract

Total knee arthroplasty (TKA) is a very common surgical treatment approach for severe osteoarthritis. Complications of TKA include loss of range of motion and prolonged analgesic requirement for pain control. Osteopathic manipulative techniques (OMT) have been utilized to address localized muscular stiffness to improve range of motion; however, limited studies directly correlate OMT and TKA recovery. This review highlights the therapeutic benefits OMT can have in the postoperative management of arthroplasty with respect to range of motion, edema, pain perception, and ability to perform activities of daily living. This review revealed the use of OMT would positively influence range of motion by manipulation of localized musculature and can result in decreased demand for analgesics. This can, in turn, shorten hospital stay and return the ability of patients to perform activities of daily living earlier than without OMT. Increased research is needed to strengthen these findings on the benefits of OMT in the postoperative management of arthroplasty.

## Introduction and background

Osteoarthritis (OA) is the most common cause of chronic disability and pain for those aged 65 or older in the United States (US) [1]. A degenerative joint disease that is caused by the breakdown of cartilage and bone, OA causes the greatest debilitation in large joints-shoulders, elbows, hips, and knees. Knee OA is particularly prevalent with the ongoing obesity epidemic as mechanical stress associated with truncal weight results in hyaline cartilage degradation of the tibiofemoral and patellofemoral joints, followed by bony remodeling and narrowing of the joint space [2]. With knee OA progression, ligamentous laxity and misalignment of the knee joint ultimately play a major role in the progression of joint structure deterioration [3]. Inflammation of the synovium and cartilage can accompany this process, leading to the hallmark symptoms of pain on ambulation and joint stiffness [4]. Treatment is often centered on symptom management, ranging from conservative measures such as lifestyle modifications and non-steroidal anti-inflammatory drugs (NSAIDs) to more invasive procedures such as corticosteroid injections and surgery [5].

Total knee arthroplasty (TKA) has become the standard approach for the management of severe knee OA when conservative, non-surgical options have failed. According to recent studies, 700,000 knee replacement surgeries are performed annually in the US and are expected to surpass 3.4 million annually by 2030 [6]. Of the patients that had undergone a knee arthroplasty, 85% reported high levels of satisfaction in the categories of patient expectations, pain, joint function, and mental factors [7]. While many patients report long-term success and satisfaction status post-TKA, the road to recovery with postoperative pain management is an area of ongoing research [8]. Joint swelling and range of motion (ROM) limitations are prevalent during the first year, with frequent visits required for care continuity, pain control, and addressing surgical sequelae [9]. Poorer outcomes are seen with those that do not address swelling and ROM restrictions. Physical therapy and analgesics are often used during post-TKA pain management, with minimal research done on alternative/complementary medicines.

Osteopathic manipulative medicine (OMM) is a branch of medicine that was founded on the philosophy that the anatomical body is interconnected to its function and physiology. Somatic dysfunction in one bodily domain can intimately affect others, resulting in pain and disability. Osteopathic manipulative treatment (OMT) is a series of techniques used to diagnose and treat somatic dysfunctions in order to restore homeostasis and return of bodily function [10]. The use of OMT is well documented in low back pain and other pain management scenarios; however, limited research exists on its use in the perioperative management of chronic knee osteoarthritis [11-14]. In addition to the obesity epidemic, the recent opioid crisis has placed greater scrutiny and interest in non-pharmaceutical methods for pain management, especially with an increasingly aging population in the US [15]. This review aims to explore the current literature on osteoarthritic pain management and how OMT can play a role in postoperative care.

## Review

Methods

**Figure 1:**
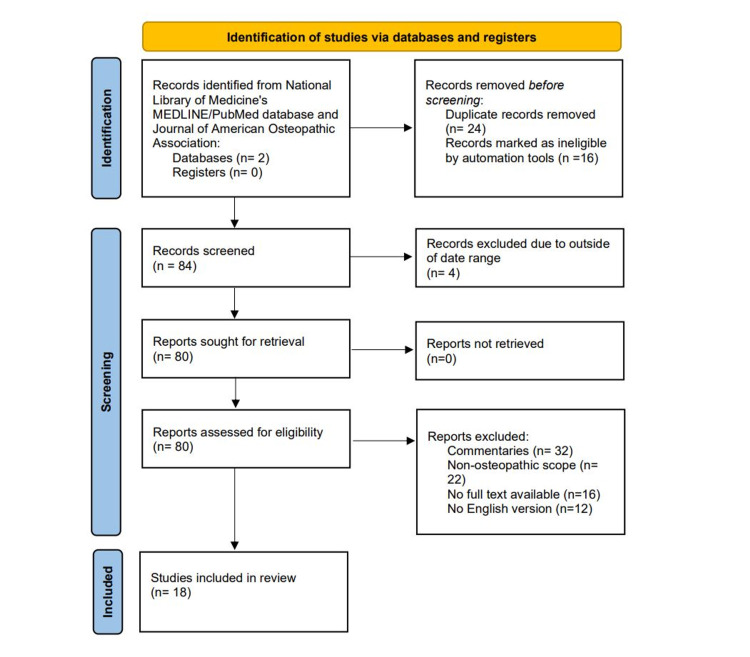
PRISMA flowchart for literature review PRISMA: Preferred Reporting Items for Systematic Reviews and Meta-Analyses

A search was conducted of the National Library of Medicine's MEDLINE/PubMed databases in addition to the Journal of American Osteopathic Association with the intent of finding all relevant articles published in English with keywords “post-operative”, “knee arthroplasty”, “total knee replacement”, and “osteopathic medicine”. All articles were accessed between September 2018 and May 2019 with qualitative data for the systematic review collected. All manuscripts that were published in English in the past 30 years were included, with the earliest in 1996. Journal publications were categorized based on study date to show progression in the field and further coded and analyzed by study type, number of participants, and conclusions to determine any overarching themes or messages. Exclusion criteria included commentaries without obvious scientific research, the non-osteopathic scope of research, inability to obtain the full text, and no English translation of the full text, with full breakdown seen in Preferred Reporting Items for Systematic Reviews and Meta-Analyses (PRISMA) guidelines (Figure 1).

Results

Eighteen studies met inclusion criteria and encompassed a wide variety, with the majority of studies performed being prospective studies (n=10), followed by case reports (n=3), cross-sectional studies (n=2), literature reviews (n=2), and case-control studies (n=1) (Table 1). Among the prospective studies, the sample sizes ranged from 43 patients to 621 patients. Two cohort studies were used with a sample size of 8325 patients. All studies were examined to evaluate at least one aspect of postsurgical complication or sequelae as the quality of the study: hospital stay, pain control, activities of daily living (ADLs), and mobility.

**Table 1:** Included publications categorized by publication date, study type, and complication examined. TKA: total knee arthroplasty, ADL: activities of daily living, OMM: osteopathic manipulative medicine

Author, Year of publication	Type of study	Participants	Study Measurements
Anouchi et al., 1996 [16]	Prospective Cohort Study	621 patients, 282 with TKA at one year and 86 at two-year follow-up	Mobility
Lizaur et al., 1997 [17]	Prospective Study	74 patients after TKA	Mobility
Slemenda et al., 1997 [1]	Cross-Sectional Prevalence Study	462 patients	Pain Control, ADL
Brittain et al., 1999 [18]	Prospective Clinical Trial	60 TKA patients, with 20 receiving OMT and 40 as a control	Hospital Stay
Jarski et al., 2000 [19]	Prospective, Match-Controlled Outcome Study	166 patients, 38 patients received OMM	Mobility, Pain Control
Scranton, 2001 [20]	Prospective Cohort Study	33 patients	Mobility, Pain Control
Sharma et al., 2001 [3]	Cross Sectional Cohort Study	237 patients; 230 completed study	ADL, Hospital Stay
Harris & Piller, 2003 [21]	Case Report/Series	Three lymphedema patients	Mobility
Ranawat et al., 2003 [22]	Prospective Observational Study	116 patients studied pre-operatively and one, three, six, and 12 months post-TKA	Hospital Stay, Pain Control
Licciardone et al., 2004 [10]	Prospective Double-Blind Study	50 patients receiving OMM after knee or hip arthroplasty	Mobility, ADL
Felson, 2006 [4]	Case Report	One patient with bilateral knee pain	Pain Control, Mobility
Gugel & Johnston, 2006 [23]	Case Report	One patient; 27-year-old, post-knee arthroscopy with subsequent somatic dysfunctions found	Mobility, ADL
Andersen et al., 2009 [24]	Prospective Cohort Study	50 knee and 50 hip arthroplasty patients	Pain Control
Garrett & Walters, 2010 [25]	Case-Control Study	280 questionnaires sent to surgeons regarding post-TKA recovery	Pain Control, ADL
Cushner et al., 2010 [9]	Prospective Observational Study	8325 post-TKA patients	Hospital Stay, ADL
Ebert et al., 2013 [26]	Prospective Randomized Controlled Trial	53 patients; 43 TKA with lymphatic post-operative treatment	Mobility, Pain Control
Pozzi et al., 2013 [8]	Systematic Review	19 studies	Hospital Stay, ADL
Schulze & Scharf, 2013 [7]	Systematic Review	25 publications from 1990-2012	Pain Control, ADL

Discussion

Current postoperative care plans for TKAs typically consist of pain control, deep vein thrombosis prevention due to prolonged leg immobility, and physical therapy in aid of regaining mobility. Pain control and deep venous thrombosis (DVT) prophylaxis are usually accomplished with medication. Physical therapy can start as early as postoperative day one, with studies showing early mobilization being associated with improved prognosis. Optimal recovery is defined as achieving 90° of knee flexion and full extension one-month post-knee arthroplasty as measured through ROM testing. Many of the studies included multiple complications, with mobility and pain control being the most common (n=9 and 9, respectively), followed by ADLs (n=8), and length of hospital stay (n=5). Of 50 surveyed patients, 52% reported moderate pain one-month postoperative TKA with concomitant use of strong opioids [24]. Other studies have indicated the prevalence of postoperative pain and stiffness in up to 10.8% of TKA patients [20]. In developing postoperative rehabilitation protocols, patients without pain tended to exhibit greater degrees in ROM, with an average of 97° in patients with pain compared to 118° without pain [24]. Of note, some patients did not regain full ROM until one year post the operation due to residual inflammation and pain, in which OMM may play a role in accelerating ROM recovery, edema alleviation, and analgesia.

ROM Recovery

OMM and OMT have been studied and used to address common pain presentations in postoperative recovery; however, limited research has been done directly following TKA [10]. In general, OMM is utilized in addressing surrounding muscle hypertonicity, encouraging local lymphatic circulation, and preventing musculoskeletal dysfunction in the postoperative period. OMM techniques are classified as active or passive based on the need for patient involvement; active techniques require patient participation, while passive techniques do not. OMM techniques are further categorized as direct or indirect, which details barrier engagement for the patient. In a direct technique, the joint being manipulated is being placed into the ROM restriction barrier while indirect techniques manipulate the joint towards freedom of motion (Table 2).

**Table 2:** Common osteopathic manipulative techniques and its usage. Of note, muscle energy and myofascial techniques have variations that are indirect; however, direct is the most common iteration.

Technique	Type	Description
Muscle Energy	Direct Active	Achieve a greater range of motion through reciprocal inhibition, by freeing the barrier of motion with patient contraction of antagonistic muscle
Myofascial	Direct Passive	Achieve relaxation of musculature and pain reduction through the Golgi tendon reflex by applying slow and constant pressure to the muscle belly
Counterstrain	Indirect Passive	Achieve muscle relaxation and pain reduction through shortening of muscles
Effleurage	Indirect Passive	Achieve an improved lymphatic drainage by applying guiding movements and gentle pressure
Lymphatic Pump	Indirect Passive	Achieve an improvement in lymphatic flow by applying rhythmic pressure.

**Figure 2:**
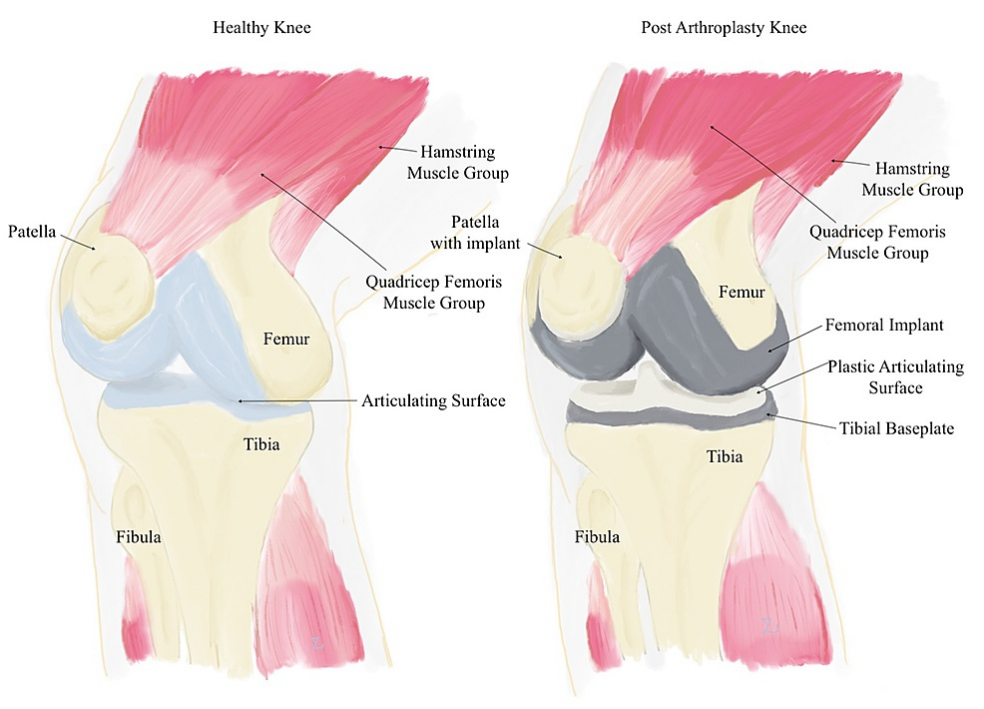
Artistic representation of normal knee anatomy compared to status post total knee replacement. Original illustration by author YaQun Zhou

Local musculature can be targeted using the theory of reciprocal inhibition, in which one muscle contracts and the antagonist muscle relaxes to accommodate this motion, leading to overall musculoskeletal and fascial relaxation. Muscle groups of interest in a total knee replacement may include the quadriceps femoris muscle group (rectus femoris, vastus lateralis, vastus medius, vastus intermedius), as well as the hamstring group muscles (semitendinosus, semimembranosus, biceps femoris) (Figure 2) [27]. While empiric studies are limited in post-surgical and post-TKA patients, it is reasonable to infer that OMM techniques can be adapted to improve function and recovery as an adjunctive measure to standard treatments.

OMT has been shown to be beneficial in a focused and localized manner as well as generalized full-body healing. Postoperative ROM restrictions are common, often due to pain, and would often lead to significant sequelae in ADL [10]. In a single-blinded controlled study done at an osteopathic teaching hospital, patients receiving OMM showed a 20% faster recovery rate in ADLs compared to control patients, which in turn resulted in quicker return to full ambulation [19]. In comparison, a randomized controlled double-blinded study done in conjunction with osteopathic medical students showed no significant difference in recovery time or overall prognosis between control and OMT groups [18]. The OMT group had various modalities performed including myofascial, counterstrain, muscle energy, high velocity-low amplitude, and craniosacral manipulation in addition to the standard rehabilitation techniques in the control group. It is important, however, to note that all OMT performed in the aforementioned study was completed by undergraduate medical students who had not completed their osteopathic education, which presented a confounding variable in determining OMT efficacy in post-TKA treatment.

Edema

Edema, or swelling caused by trapped extracellular fluid in body tissue, is a common sequela reported in TKA patients, particularly in infrapatellar structures. Postoperative edema can have numerous etiologies ranging from anatomical sources such as surgical trauma to lymphatic channels to physiological reasons such as decreased movement and pain during recovery. Basic OMT manual lymphatic drainage (MLD) techniques include the use of rhythmic alternating pressure, in which pressure is applied in the direction of fluid movement and fascial restrictions are adjusted to move stagnant flow [21]. A complication occasionally seen in post-TKA edema is ischemia, in which the built-up fluid creates a physical barrier that restricts blood flow to the distal limb. OMT with manual lymphatic drainage has been shown to decrease the edema by stimulating and assisting the drainage of extra fluid in the lower extremities through clearance of proximal restrictions [28]. Thoracic outlet release, diaphragmatic inhibition, pelvic diaphragm release, and effleurage have been shown to aid in the re-uptake of edematous tissues into the central lymphatic system [29,30]. Pedal pump and popliteal diaphragm release should be done with caution as excess movement distally can adversely affect operation site healing [11,31]. Limited studies have been done, however, with OMT in post-TKA patients, with preliminary results showing no significant differences in MLD versus control patients [26].

Analgesia and Daily Function

Pain and its control are often challenging components to tackle in the post-TKA recovery period and often limit a patient’s ability to participate in physical therapy and return to daily activities. OMT has been shown to shorten hospital length of stays and reduce dependency/duration of opioid analgesics, which leads to an overall lifestyle improvement. Prior studies have noted that with regaining preoperative ROM, those in pain tend to have an increased number of postoperative office visits compared to those with resolving pain. Clinical trials with OMT in this recovery period have been shown to be associated with shorter rehabilitation periods compared to standard control groups and had earlier return to activities such as stair ambulation [19]. Functional Independence Measurement (FIM) was designed during clinical trials for OMT to measure one's functional independence through a seven-point rating scale in various daily activities [18]. In comparing OMT and control groups, both groups showed improvement of total FIM post-operation as a baseline; however, no statistically significant findings were observed. Nevertheless, when analyzing FIM sub-categories, the OMT group had greater stair climbing mobility and ambulation, both of which were correlated to greater patient satisfaction and patient recovery [10]. With faster recovery, TKA patients return to daily life sooner and with fewer associated sequelae.

Future outlook

Based on the 18 studies reviewed, there is insufficient evidence to determine the true role of OMM/OMT in post-TKA patients. While the benefit of OMM in its application to post-TKA patients can be inferred, many studies did not explicitly include OMM or OMT as a treatment modality, thus limiting a comparative approach to conventional or standardized postoperative treatments. Despite the inclusion of multiple prospective studies, a more definitive clinical trial with control is needed for comparison. OMM/OMT protocols tended to be subjective and personalized, in which reproducibility and the use of double-blinded studies would be challenging. Objective measures for subjective categories such as patient satisfaction tend to produce more variable results, which complicates generalizations. Future research can develop objective measures that assess the improvement or wellness of the patients post-TKA after receiving OMM. Further investigation can also be done with OMT in other common joint arthroplasties such as hips and shoulders, which can expand the role of OMT in the postoperative patient.

## Conclusions

OMM can potentially provide therapeutic benefits for patients following TKA by inhibiting common pathological processes common in the postoperative period, including muscle hypertonicity, edema, and musculoskeletal dysfunction. OMT, such as muscle energy, myofascial release, strain and counterstrain, effleurage, and lymphatic pump, can provide relief, especially in regard to edema, pain, and restricted ROM, subsequently increasing mobility, which can correlate with decreased length of hospital stay, improved pain control, and greater capability of achieving ADLs. In the current opioid epidemic, alternatives to medical therapy can assist patients in their return to daily activity performance and, therefore, yield positive influences on the mental health of patients postoperatively. While studies provide evidence of the potential benefits of OMT in the postoperative management of arthroplasty, increased research on the benefits of OMT is needed to strengthen these findings. The limited studies included in this review emphasize the importance of additional research in the field of OMM.
